# Supplementary material for: Temperature-Dependent Structural Evolution of Ruddlesden–Popper Bilayer Nickelate La3Ni2O7
Source: Inorg Chem. 2025 Jan 10;64(2):828–34. doi: 10.1021/acs.inorgchem.4c03042 (PMC11752513; doi:10.1021/acs.inorgchem.4c03042)
Supplement: Supplementary file 1 — ic4c03042_si_001.pdf [file ic4c03042_si_001.pdf]

Supporting Information

Temperature-dependent Structural Evolution of Ruddlesden–Popper Bilayer Nickelate $La_3Ni_2O_7$

Haozhe Wang[1], Haidong Zhou[2], Weiwei Xie[1]*

1. Department of Chemistry, Michigan State University, East Lansing, MI, 48824, USA
2. Department of Physics and Astronomy, University of Tennessee, Knoxville, TN, 37996, USA

* Email: xieweiwe@msu.edu

Table of Contents

Figure S1. Reciprocal lattice planes of LNO-2222 at 120 K, 160 K, and 200 K S2
Figure S2. Reciprocal lattice planes of LNO-2222 at 240 K, 320 K, and 360 K S3
Figure S3. Temperature-dependent structure evolution of LNO-2222....S4

Table S1. Crystal data and structure refinement of LNO-2222 at 120 K S5
Table S2. Atomic coordinates and equivalent isotropic atomic displacement parameters at 120 K S5
Table S3. Crystal data and structure refinement of LNO-2222 at 160 K S6
Table S4. Atomic coordinates and equivalent isotropic atomic displacement parameters at 160 K S6
Table S5. Crystal data and structure refinement of LNO-2222 at 200 K S7
Table S6. Atomic coordinates and equivalent isotropic atomic displacement parameters at 200 K S7
Table S7. Crystal data and structure refinement of LNO-2222 at 240 K S8
Table S8. Atomic coordinates and equivalent isotropic atomic displacement parameters at 240 K S8
Table S9. Crystal data and structure refinement of LNO-2222 at 320 K S9
Table S10. Atomic coordinates and equivalent isotropic atomic displacement parameters at 320 K S9
Table S11. Crystal data and structure refinement of LNO-2222 at 360 K S10
Table S12. Atomic coordinates and equivalent isotropic atomic displacement parameters at 360 K S10
Table S13. Crystal data and structure refinement of LNO-2222 at 400 K S11
Table S14. Atomic coordinates and equivalent isotropic atomic displacement parameters at 400 K S11

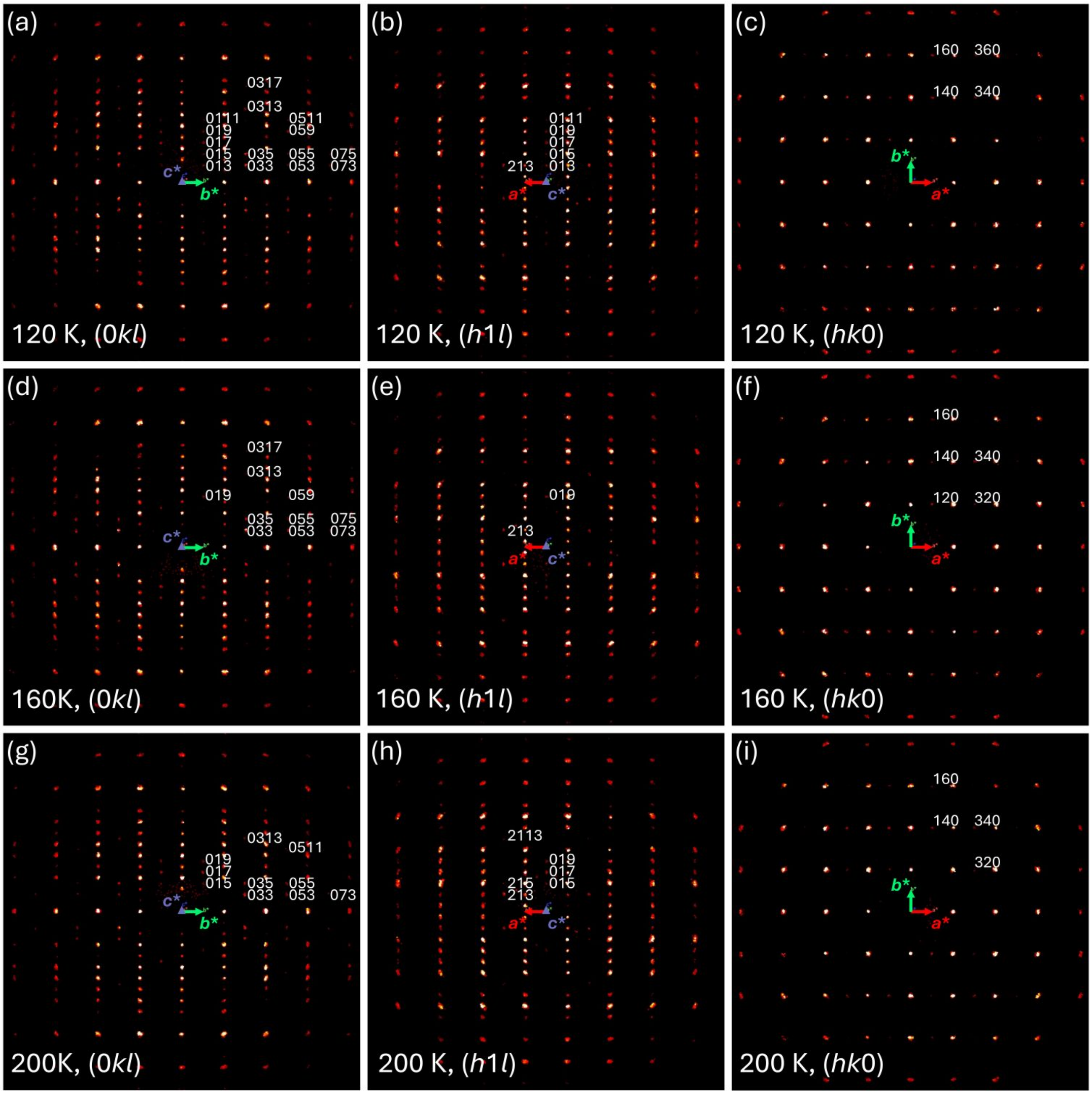

Figure S1. Reciprocal lattice planes of LNO-2222. **(a–c)** (0*kl*), (*h*1*l*) and (*hk*0) planes at 120 K. **(d–f)** (0*kl*), (*h*1*l*) and (*hk*0) planes at 160 K. **(g–i)** (0*kl*), (*h*1*l*) and (*hk*0) planes at 200 K. Laue symmetry *mmm* has been applied in the regeneration of these (*hkl*) planes.

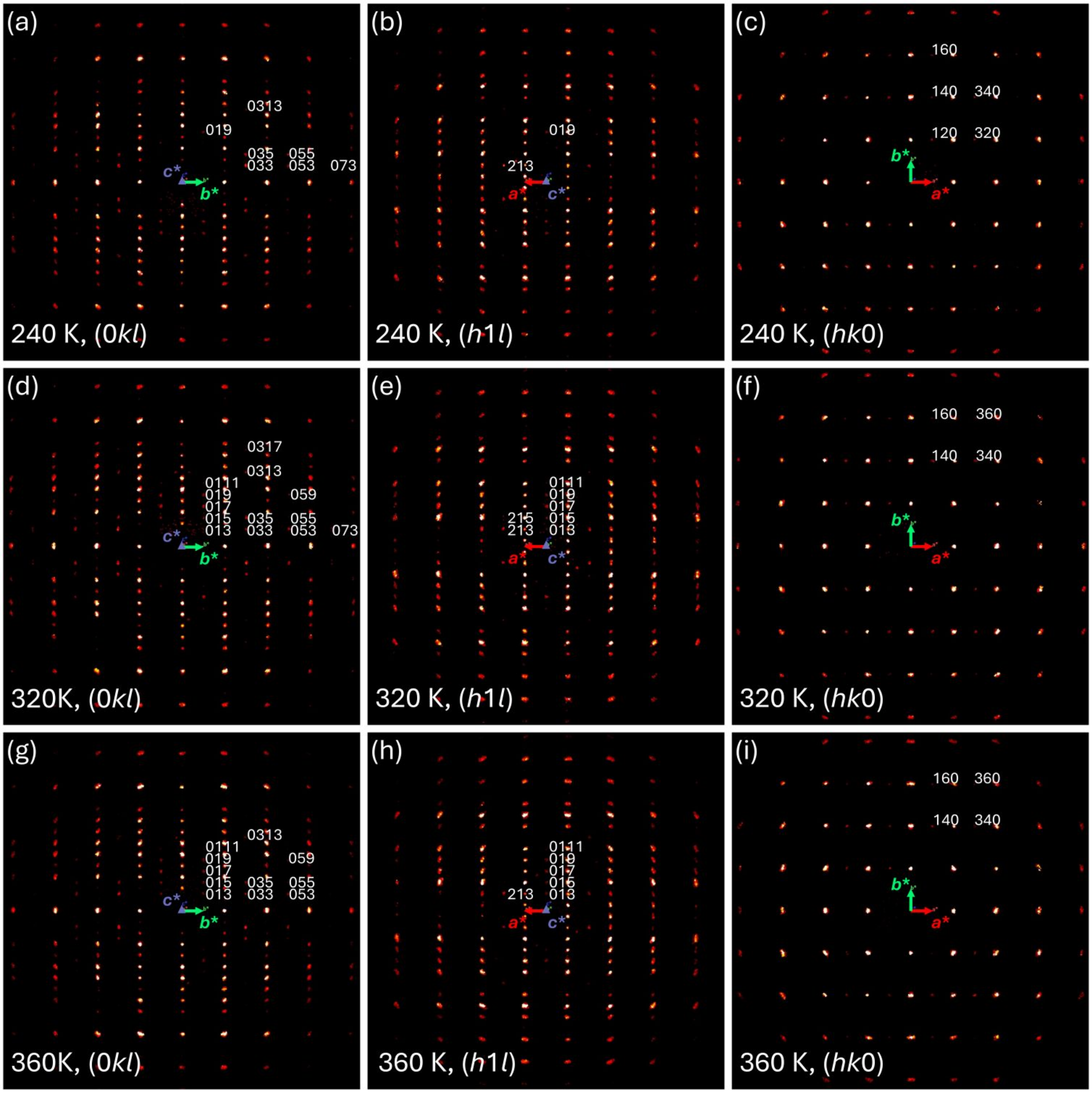

Figure S2. Reciprocal lattice planes of LNO-2222. **(a–c)** (0*kl*), (*h*1*l*) and (*hk*0) planes at 240 K. **(d–f)** (0*kl*), (*h*1*l*) and (*hk*0) planes at 320 K. **(g–i)** (0*kl*), (*h*1*l*) and (*hk*0) planes at 360 K. Laue symmetry *mmm* has been applied in the regeneration of these (*hkl*) planes.

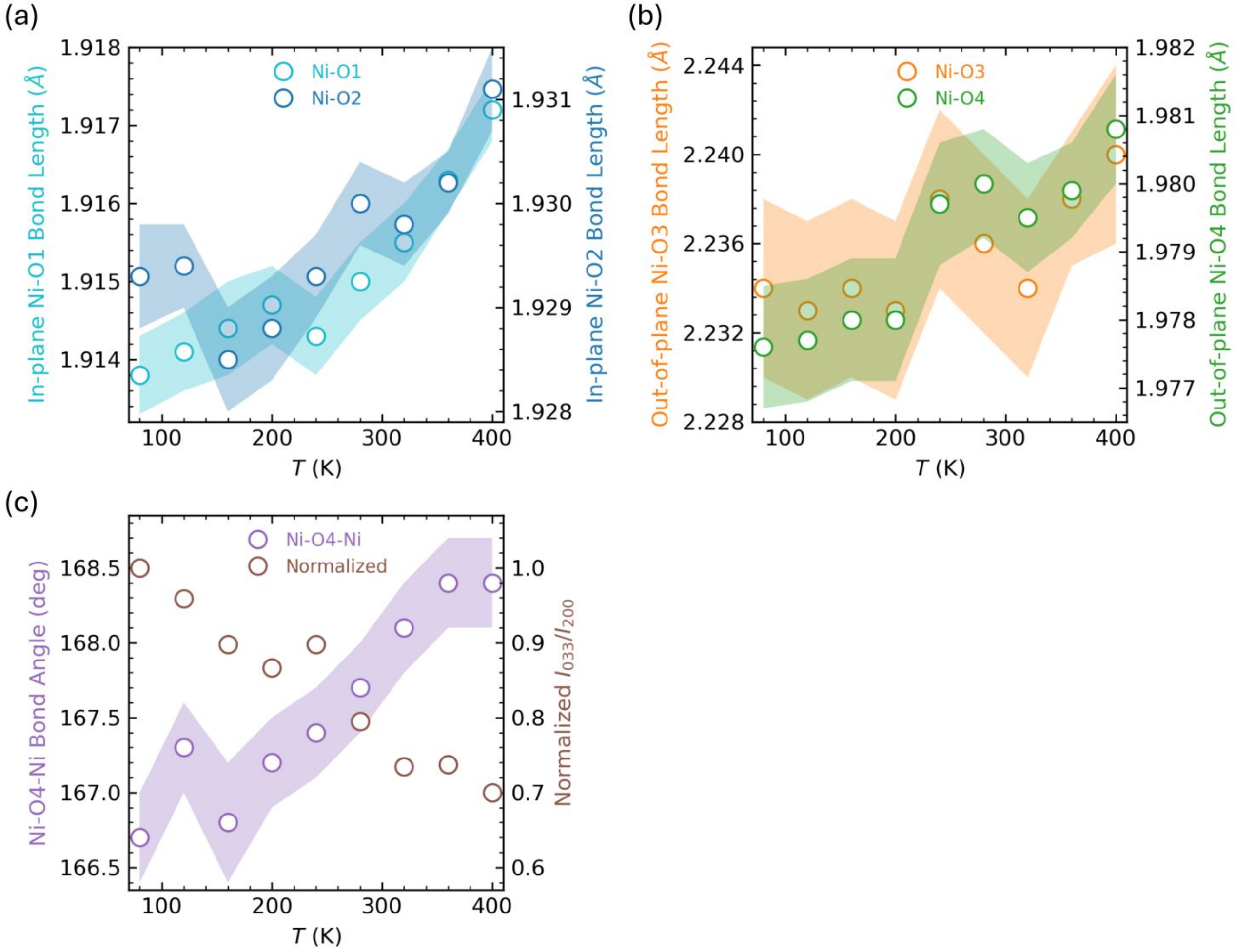

Figure S3. Temperature-dependent structure evolution of LNO-2222. **(a)** In-plane Ni–O1 and Ni–O2 bond length. **(b)** Out-of-plane Ni–O3 and Ni–O4 bond length. **(c)** Out-of-plane Ni–O4–Ni bond angle and the observed intensity ratio of the 033 and 200 reflections, normalized to 80 K. Error bars are indicated by color filling.

Table S1. Crystal data and structure refinement of LNO-2222 at 120 K.

**Chemical formula**	**$La_3Ni_2O_7$-2222**
Temperature	120(2) K
Formula weight	646.15 g/mol
Space group	*Amam*
Unit cell dimensions	a = 5.37971(16) Å
	b = 5.44838(15) Å
	c = 20.4948(6) Å
Volume	600.72(3) $Å^3$
Z	4
Density (calculated)	7.145 g/cm^3
Absorption coefficient	26.995 mm^{-1}
$F(000)$	1132
θ range	3.87 to 40.69°
Reflections collected	18733
Independent reflections	1051 [R_{int} = 0.0664]
Refinement method	Full-matrix least-squares on F^2
Data / restraints / parameters	1051 / 0 / 37
Final R indices	R_1 (I>2σ(I)) = 0.0279; wR_2 (I>2σ(I)) = 0.0695
	R_1 (all) = 0.0340; wR_2 (all) = 0.0726
Largest diff. peak and hole	+6.077 e/$Å^3$ and –1.437 e/$Å^3$
R.M.S. deviation from mean	0.493 e/$Å^3$
Goodness-of-fit on F^2	1.184

Table S2. Atomic coordinates and equivalent isotropic atomic displacement parameters ($Å^2$) of LNO-2222 at 120 K. U_{eq} is defined as one third of the trace of the orthogonalized U_{ij} tensor.

	**Wyck.**	***x***	***y***	***z***	**Occ.**	**U_{eq}**
**La_1**	4*c*	1/4	0.24918(5)	0	1	0.00407(8)
**La_2**	8*g*	1/4	0.24139(3)	0.17976(2)	1	0.00375(7)
**Ni**	8*g*	1/4	0.74747(8)	0.90410(3)	1	0.00299(10)
**O_1**	4*c*	1/4	0.7072(9)	0	1	0.0081(7)
**O_2**	8*g*	3/4	0.2142(6)	0.20436(16)	1	0.0077(5)
**O_3**	8*e*	0	0	0.91113(16)	1	0.0067(5)
**O_4**	8*e*	0	1/2	0.89470(17)	1	0.0072(5)

Table S3. Crystal data and structure refinement of LNO-2222 at 160 K.

**Chemical formula**	**$La_3Ni_2O_7$-2222**
Temperature	160(2) K
Formula weight	646.15 g/mol
Space group	*Amam*
Unit cell dimensions	a = 5.38099(18) Å
	b = 5.44670(18) Å
	c = 20.4940(7) Å
Volume	600.65(3) $Å^3$
Z	4
Density (calculated)	7.145 g/cm^3
Absorption coefficient	26.980 mm^{-1}
$F(000)$	1132
θ range	3.87 to 40.69°
Reflections collected	18669
Independent reflections	1052 [R_{int} = 0.0708]
Refinement method	Full-matrix least-squares on F^2
Data / restraints / parameters	1052 / 0 / 37
Final R indices	R_1 (I>2σ(I)) = 0.0302; wR_2 (I>2σ(I)) = 0.0713
	R_1 (all) = 0.0379; wR_2 (all) = 0.0748
Largest diff. peak and hole	+5.523 e/$Å^3$ and –1.806 e/$Å^3$
R.M.S. deviation from mean	0.493 e/$Å^3$
Goodness-of-fit on F^2	1.105

Table S4. Atomic coordinates and equivalent isotropic atomic displacement parameters ($Å^2$) of LNO-2222 at 160 K. U_{eq} is defined as one third of the trace of the orthogonalized U_{ij} tensor.

	**Wyck.**	***x***	***y***	***z***	**Occ.**	**U_{eq}**
**La_1**	4*c*	1/4	0.24913(5)	0	1	0.00467(8)
**La_2**	8*g*	1/4	0.24157(4)	0.17977(2)	1	0.00420(7)
**Ni**	8*g*	1/4	0.74758(9)	0.90413(3)	1	0.00328(10)
**O_1**	8*g*	1/4	0.7850(7)	0.79560(17)	1	0.0085(5)
**O_2**	8*e*	0	0	0.91090(17)	1	0.0072(6)
**O_3**	8*e*	0	1/2	0.10523(18)	1	0.0078(5)
**O_4**	4*c*	1/4	0.7057(10)	0	1	0.0081(7)

Table S5. Crystal data and structure refinement of LNO-2222 at 200 K.

**Chemical formula**	**$La_3Ni_2O_7$-2222**
Temperature	200(2) K
Formula weight	646.15 g/mol
Space group	*Amam*
Unit cell dimensions	a = 5.38379(19) Å
	b = 5.44572(19) Å
	c = 20.5041(7) Å
Volume	601.15(4) Å^3
Z	4
Density (calculated)	7.139 g/cm^3
Absorption coefficient	26.950 mm^{-1}
F(000)	1132
θ range	3.87 to 40.67°
Reflections collected	18561
Independent reflections	1050 [R_{int} = 0.0849]
Refinement method	Full-matrix least-squares on F^2
Data / restraints / parameters	1050 / 0 / 37
Final R indices	R_1 (I>2σ(I)) = 0.0300; wR_2 (I>2σ(I)) = 0.0705
	R_1 (all) = 0.0391; wR_2 (all) = 0.0747
Largest diff. peak and hole	+5.868 e/Å^3 and –2.042 e/Å^3
R.M.S. deviation from mean	0.527 e/Å^3
Goodness-of-fit on F^2	1.091

Table S6. Atomic coordinates and equivalent isotropic atomic displacement parameters (Å^2) of LNO-2222 at 200 K. U_{eq} is defined as one third of the trace of the orthogonalized U_{ij} tensor.

	**Wyck.**	***x***	***y***	***z***	**Occ.**	**U_{eq}**
**La_1**	4*c*	1/4	0.24927(6)	0	1	0.00547(8)
**La_2**	8*g*	1/4	0.24177(4)	0.17978(2)	1	0.00490(7)
**Ni**	8*g*	1/4	0.74755(8)	0.90413(3)	1	0.00368(10)
**O_1**	8*g*	1/4	0.7847(7)	0.79568(17)	1	0.0095(6)
**O_2**	8*e*	0	0	0.91077(17)	1	0.0087(6)
**O_3**	8*e*	0	1/2	0.10525(18)	1	0.0084(6)
**O_4**	4*c*	1/4	0.7072(9)	0	1	0.0086(7)

Table S7. Crystal data and structure refinement of LNO-2222 at 240 K.

**Chemical formula**	**$La_3Ni_2O_7$-2222**
Temperature	240(2) K
Formula weight	646.15 g/mol
Space group	*Amam*
Unit cell dimensions	a = 5.38598(18) Å
	b = 5.44615(17) Å
	c = 20.5184(6) Å
Volume	601.86(3) Å^3
Z	4
Density (calculated)	7.131 g/cm^3
Absorption coefficient	26.926 mm^{-1}
F(000)	1132
θ range	3.87 to 40.66°
Reflections collected	18597
Independent reflections	1053 [R_{int} = 0.0870]
Refinement method	Full-matrix least-squares on F^2
Data / restraints / parameters	1053 / 0 / 37
Final R indices	R_1 (I>2σ(I)) = 0.0290; wR_2 (I>2σ(I)) = 0.0733
	R_1 (all) = 0.0377; wR_2 (all) = 0.0779
Largest diff. peak and hole	+5.754 e/Å^3 and –1.901 e/Å^3
R.M.S. deviation from mean	0.498 e/Å^3
Goodness-of-fit on F^2	1.118

Table S8. Atomic coordinates and equivalent isotropic atomic displacement parameters (Å^2) of LNO-2222 at 240 K. U_{eq} is defined as one third of the trace of the orthogonalized U_{ij} tensor.

	**Wyck.**	***x***	***y***	***z***	**Occ.**	**U_{eq}**
**La_1**	4*c*	1/4	0.24931(5)	0	1	0.00632(8)
**La_2**	8*g*	1/4	0.24196(3)	0.17979(2)	1	0.00557(7)
**Ni**	8*g*	1/4	0.74751(8)	0.90410(3)	1	0.00410(10)
**O_1**	8*g*	1/4	0.7845(6)	0.79546(16)	1	0.0108(6)
**O_2**	8*e*	0	0	0.91071(16)	1	0.0087(6)
**O_3**	8*e*	0	1/2	0.10494(18)	1	0.0094(5)
**O_4**	4*c*	1/4	0.7075(9)	0	1	0.0105(7)

Table S9. Crystal data and structure refinement of LNO-2222 at 320 K.

**Chemical formula**	**$La_3Ni_2O_7$-2222**
Temperature	320(2) K
Formula weight	646.15 g/mol
Space group	*Amam*
Unit cell dimensions	a = 5.39364(20) Å
	b = 5.44680(19) Å
	c = 20.5372(7) Å
Volume	603.34(4) $Å^3$
Z	4
Density (calculated)	7.113 g/cm^3
Absorption coefficient	26.870 mm^{-1}
$F(000)$	1132
θ range	3.87 to 40.70°
Reflections collected	18658
Independent reflections	1057 [R_{int} = 0.0640]
Refinement method	Full-matrix least-squares on F^2
Data / restraints / parameters	1057 / 0 / 37
Final R indices	R_1 (I>2σ(I)) = 0.0267; wR_2 (I>2σ(I)) = 0.0661
	R_1 (all) = 0.0352; wR_2 (all) = 0.0697
Largest diff. peak and hole	+5.368 e/$Å^3$ and –1.374 e/$Å^3$
R.M.S. deviation from mean	0.448 e/$Å^3$
Goodness-of-fit on F^2	1.114

Table S10. Atomic coordinates and equivalent isotropic atomic displacement parameters ($Å^2$) of LNO-2222 at 320 K. U_{eq} is defined as one third of the trace of the orthogonalized U_{ij} tensor.

	**Wyck.**	***x***	***y***	***z***	**Occ.**	**U_{eq}**
**La_1**	4*c*	1/4	0.24944(5)	0	1	0.00782(7)
**La_2**	8*g*	1/4	0.24232(3)	0.17980(2)	1	0.00673(7)
**Ni**	8*g*	1/4	0.74762(7)	0.90413(3)	1	0.00490(9)
**O_1**	4*c*	1/4	0.7101(8)	0	1	0.0122(7)
**O_2**	8*e*	0	0	0.91036(15)	1	0.0116(5)
**O_3**	8*e*	0	1/2	0.89542(16)	1	0.0124(5)
**O_4**	8*g*	1/4	0.7824(6)	0.79572(16)	1	0.0137(6)

Table S11. Crystal data and structure refinement of LNO-2222 at 360 K.

**Chemical formula**	**$La_3Ni_2O_7$-2222**
Temperature	360(2) K
Formula weight	646.15 g/mol
Space group	*Amam*
Unit cell dimensions	a = 5.39743(18) Å
	b = 5.44856(18) Å
	c = 20.5506(6) Å
Volume	604.36(3) Å^3
Z	4
Density (calculated)	7.102 g/cm^3
Absorption coefficient	26.809 mm^{-1}
F(000)	1132
θ range	3.87 to 40.81°
Reflections collected	18236
Independent reflections	1057 [R_{int} = 0.0516]
Refinement method	Full-matrix least-squares on F^2
Data / restraints / parameters	1057 / 0 / 37
Final R indices	R_1 (I>2σ(I)) = 0.0254; wR_2 (I>2σ(I)) = 0.0630
	R_1 (all) = 0.0327; wR_2 (all) = 0.0659
Largest diff. peak and hole	+5.478 e/Å^{-3} and –1.303 e/Å^{-3}
R.M.S. deviation from mean	0.417 e/Å^{-3}
Goodness-of-fit on F^2	1.112

Table S12. Atomic coordinates and equivalent isotropic atomic displacement parameters (Å^2) of LNO-2222 at 360 K. U_{eq} is defined as one third of the trace of the orthogonalized U_{ij} tensor.

	**Wyck.**	***x***	***y***	***z***	**Occ.**	**U_{eq}**
**La_1**	4*c*	1/4	0.24956(4)	0	1	0.00854(7)
**La_2**	8*g*	1/4	0.24251(3)	0.17980(2)	1	0.00734(6)
**Ni**	8*g*	1/4	0.74767(6)	0.90415(2)	1	0.00523(9)
**O_1**	8*e*	1/4	0.7110(8)	0	1	0.0133(6)
**O_2**	8*e*	0	0	0.91007(14)	1	0.0125(5)
**O_3**	8*g*	0	1/2	0.89565(16)	1	0.0133(5)
**O_4**	4*c*	1/4	0.7811(5)	0.79561(14)	1	0.0138(5)

Table S13. Crystal data and structure refinement of LNO-2222 at 400 K.

**Chemical formula**	**$La_3Ni_2O_7$-2222**
Temperature	400(2) K
Formula weight	646.15 g/mol
Space group	*Amam*
Unit cell dimensions	a = 5.4020(2) Å
	b = 5.4511(2) Å
	c = 20.5660(7) Å
Volume	605.60(4) $Å^3$
Z	4
Density (calculated)	7.087 g/cm^3
Absorption coefficient	26.728 mm^{-1}
$F(000)$	1132
θ range	3.87 to 40.78°
Reflections collected	18469
Independent reflections	1059 [R_{int} = 0.0664]
Refinement method	Full-matrix least-squares on F^2
Data / restraints / parameters	1059 / 0 / 37
Final R indices	R_1 (I>2σ(I)) = 0.0273; wR_2 (I>2σ(I)) = 0.0685
	R_1 (all) = 0.0363; wR_2 (all) = 0.0727
Largest diff. peak and hole	+5.997 e/$Å^{-3}$ and –1.109 e/$Å^{-3}$
R.M.S. deviation from mean	0.448 e/$Å^{-3}$
Goodness-of-fit on F^2	1.121

Table S14. Atomic coordinates and equivalent isotropic atomic displacement parameters ($Å^2$) of LNO-2222 at 400 K. U_{eq} is defined as one third of the trace of the orthogonalized U_{ij} tensor.

	**Wyck.**	***x***	***y***	***z***	**Occ.**	**U_{eq}**
**La_1**	4*c*	1/4	0.24964(4)	0	1	0.00955(8)
**La_2**	8*g*	1/4	0.24277(3)	0.17980(2)	1	0.00808(7)
**Ni**	8*g*	1/4	0.74769(6)	0.90418(3)	1	0.00579(9)
**O_1**	4*c*	1/4	0.7109(8)	0	1	0.0136(7)
**O_2**	8*g*	1/4	0.7811(5)	0.79561(15)	1	0.0150(5)
**O_3**	8*e*	0	0	0.90988(15)	1	0.0136(5)
**O_4**	8*e*	0	1/2	0.89594(16)	1	0.0146(5)
